# Medical Society of the County of Erie

**Published:** 1901-02

**Authors:** Franklin C. Gram


### Society Proceedings.

### MEDICAL SOCIETY OF THE COUNTY OF ERIE.

EIGHTIETH ANNUAL MEETING, JANUARY 8, 1901.

REPORTED BY FRANKLIN C. GRAM, M. D., Secretary.

THE president, Dr. E. H. BALLOU, of Gardenville, called the meeting to order at 10 a.m., January 8, 1901, in the Y.M.C.A., Parlors, where a large number of the members had assembled.

After the minutes of the previous meeting had been adopted the president appointed a nominating committee, consisting of Drs. Wm. C. Krauss, Irving W. Potter and W. D. Bidaman.

Dr. J. J. WALSH, of the committee on membership, reported favorably on the following-named applicants, who were thereupon elected to membership in the society: Drs. Henry C. Lapp, of Clarence; Geo. N. Jack, of Depew; Wm. C. Jolls, of Orchard Park; Frederick H. Ehinger, of Ebenezer; Prescott LeBreton, Grosvenor R. Trowbridge and James J. Mooney, of Buffalo.

The president then called Dr. Coakley to the chair and delivered his annual address, entitled, A Country Health Officer and the Board of Health. (See page 488.)

Dr. J. B. COAKLEY, chairman of the board of censors, next submitted the annual report of the board as follows:

*To the Medical Society of the County of Erie:*

Since the last report made to the society in June, 1900, the censors have continued their work of investigating cases of alleged illegal practitioners of medicine and surgery. There have been a number of such cases in which persons violating the law have done so under a mistake, and, having discontinued so doing, your board has deemed it unnecessary to institute any prosecutions.

In the case of William Carpenter, who is already under indictment for illegally practising medicine, a second indictment on what seemed to your board and the grand jury and district attorney amply sufficient evidence to warrant a conviction was found. The case was tried ably and well on the part of the prosecution, Carpenter having practically no defense, and the only appeal which was made in his behalf by his counsel was a claim that if he was convicted and fined, the fine instead of going to the county treasurer would be paid over to this society for its own private purposes. Of course, the court charged the jury that this was no concern of theirs, but the jury promptly rendered a verdict of not guilty. Some of the jurors have since informed your counsel that it was their unanimous opinion that they would not give a verdict in any case of this kind convicting the accused, while the law stood as it does at present. We have, therefore, come to the conclusion that it would be well for the law to be amended, either by striking out this provision for the payment of the fine to the medical society entirely, or by modifying it so that one-half or one fourth only should be paid to this society.

Our counsel is in correspondence with the Hon. Robert W. Taylor, of New York, counsel for the Medical Society of the County of New York, in regard to several amendments to the medical law, which would have been adopted last winter but for the fact that the statutory revision commission was abolished. We respectfully urge that he be authorised to arrange, if possible, for the coöperation of the New York Medical Society in amending the law in reference to the payment of the fine as above indicated.

In the case of Arthur de Collard, who advertised in the Buffalo *Express* as "Dr. de Collard," and under the guise of massage treatment, was diagnosticating cases and giving some ointments and, in one instance, iron tablets, your board obtained affidavits and obtained a warrant of arrest for Collard from the police justice. On the trial Collard proved by a man named Brooks, part of whose house he occupies, that he Brooks, wrote the advertisement and put in the title of "Dr." without Collard's consent, and that the said advertisement had been discontinued as soon as he was arrested. He claimed also that his treatment was nothing but ordinary massage treatment. The police justice declined to hold him for the grand jury. There is but little doubt that he imposes upon the credulity of the public and his patients more or less, and your board has under consideration the advisability of presenting his case to the grand jury at its next session.

While it may appear that the prosecutions which your board of censors have instituted have not resulted in convictions, it is undoubtedly true that the publicity given to these prosecutions, and the fact that it is known that your board makes rigid investigations and attempts to bring to punishment offenders against the medical law, has a salutary effect in preventing violations of this law.

In conclusion, this board respectfully recommends that the Hon. Tracy C. Becker be continued as counsel on the same terms as heretofore. We also recommend that this board be given authority to employ such detectives and other agents as may be required for the performance of its duties, and to draw upon the treasurer as heretofore for their reasonable compensation.

Respectfully submitted,

J. B. Coakley, *Chairman.*

Henry R. Hopkins.

Thomas F. Dwyer.

Francis E. Fronczak.

Irving W. Potter.

The report was adopted and the thanks of the society extended to the board.

Dr. Wm. C. Krauss, of the committee on nominations, submitted the following report: For president, Dr. Wm. C. Phelps, Buffalo; for vice-president, Dr. Walden M. Ward, North Collins; for secretary, Dr. Franklin C. Gram; for treasurer, Dr. Edward Clark; for librarian, Dr. Wm. C. Callanan; for board of censors, Dr. J. B. Coakley, chairman; and Drs. Henry R. Hopkins, Irving W. Potter, F. E. Fronczak, and Henry Lapp, of Clarence.

For delegates to the Medical Society of the State of New York: Drs. Justin G. Thompson, of Angola; John D. McPherson, of Akron; Walter D. Greene, Henry D. Ingraham, Irving M. Snow, Wm. G. Bissell, A. L. Benedict and N. W. Wilson, of Buffalo.

For Alternates: Drs. E. H. Ballou, Gardenville; R. S. Meyers, Clarence Center; C. E. Congdon, Dewitt H. Sherman, Chauncy P. Smith, Sidney A. Dunham, Grosvenor R. Trowbridge and Geo. F. Cott, of Buffalo.

In presenting the list of alternates, Dr. Krauss stated that the number of members who were desirous of becoming delegates was far in excess of the vacancies, as only one delegate could be sent for each assembly district; but it sometimes happens that a regular delegate is unable to attend, and in order not to lose representation this plan was proposed. He moved the adoption of the report and that the secretary be instructed to cast the vote of the society for the nominees.

This motion was adopted and the secretary cast the ballot as directed. The president then declared the officers, as reported by the committee, duly elected for the ensuing year.

Dr. A. L. Benedict, of the special committee, appointed at the semi-annual meeting, made the following report:

*To the Medical Society of the County of Erie, State of New York:*

Your committee, appointed to consider means for harmonising the regular profession of medicine in this state, and for securing recognition in the American Medical Association for members of the profession in affiliation with the Medical Society of the State of New York, would recommend the adoption of the following resolution:

Whereas, we, the members of the Medical Society of the County of Erie, deprecate all schism in the medical profession and, particularly, the double system of state and county organisation, at present separating regular physicians who are practically in harmony, both as regards methods of practice and principles of ethics;

Whereas, we believe the first step in securing a reunion of the medical profession of the State of New York consists in obtaining for the Medical Society of the State of New York and its dependent county societies, representation and membership in the American Medical Association;

Be it *Resolved*, That we petition the Medical Society of the State of New York to apply for representation in the American Medical Association;

Be it further *Resolved*, That a copy of the resolutions be transmitted to the secretary of the Medical Society of the State of New York and of each County Medical Society of New York State.

A. L. Benedict, *Chairman.*
J. B. Coakley
Eli H. Long.

Buffalo, January 8, 1901.

Dr. Henry R. Hopkins, of the committee, presented the following minority report:

Whereas, it is the deliberate judgment of the Medical Society of the County of Erie, that the medical profession of the State of New York will be more efficiently and intelligently organised for the promotion of the knowledge of the healing art, and the preservation of the public health, when there shall be one medical society, and in each county, one county medical society—which several societies shall include in this membership all persons authorised by law to practise physic and surgery in the State of New York; therefore

*Resolved*, That all by-laws, codes, regulations and usages contrary or repugnant to the foregoing principles, be and the same hereby are declared to be antagonistic to the best interests of the medical profession and inimical to the public health.

*Resolved*, That the Medical Society of the County of Erie through its president and secretary invite all persons duly licensed by law to practise physic and surgery and residing in the County of Erie, State of New York, to join the Medical Society of the County of Erie.

*Resolved*, That these resolutions duly attested be transmitted to the Medical Society of the State of New York, and to the several county medical societies of the State.

Henry R. Hopkins.

Buffalo, January 8, 1901.

Dr. Hopkins did not consider it advisable to dispose of either report at the present meeting. Both contained suggestions which required careful consideration and this could be better done by giving the members an opportunity to read the reports with the regular proceedings. He moved that both reports lie on the table until the June meeting.

Dr. Benedict advocated an immediate disposition, but Dr. Hopkins's motion prevailed, whereupon both reports were received and filed.

The treasurer, Dr. Edward Clark, submitted his annual report. The receipts were $460.76, and the expenditures, $415.18, leaving a balance of $45.58. The separate accounts and vouchers were submitted to Drs. Fronczak and Lapp for audit, who later reported finding them correct.

Dr. Lucien Howe presented the following:

*Resolved*, That a committee of three be appointed whose duty it shall be to select not more than 100 delegates from the Medical Society of the County of Erie, to the Medical Association of Central New York, and the members thus selected by that committee shall be considered as delegates from this society to that association.

The resolution was adopted and the chair appointed as such committee Drs. A. A. Jones, Lucien Howe and Wm. C. Krauss.

Dr. Charles E. Congdon called attention to the difficulty experienced by physicians in collecting accounts. The physician usually comes last in the consideration of just debts. Laborers and mechanics are given preference under the law, having the privilege of enforcing payment of judgments under body executions by which a delinquent may be imprisoned until a debt is paid, but professional men, whose services are required in the saving of human life, have no protection, except by the usual methods of enforcing claims. He cited instances to support his assertions and believed that in these days when all classes organise for mutual protection the physician should be in line and awake to his own interests.

He advocated the formation of a union for this purpose, and as such an innovation requires considerable time and study as to the best methods of procedure, he moved that a committee of three be appointed to draft plans and present them to the society at its next regular meeting.

This resolution was adopted and the chair named Drs. Congdon, W. G. Bissell and D. J. Constantine as such committee.

Dr. J. H. Pryor gave a brief review of legislation in reference to tuberculosis in this state. He pointed out the main features of what had been accomplished and what remained to be done.

At the conclusion of his remarks Dr. H. R. Hopkins offered the following resolution, which was adopted:

*Resolved*, That the Medical Society of the County of Erie hereby affirms its earnest belief in the truth of the infectiousness, the preventability, the curability of consumption, and therefore commends to the medical societies of the several counties of the State of New York, and to the Medical Society of the State of New York, the proposition for a state sanatorium for the cure of incipient consumptives and of municipal hospitals for the treatment of advanced cases of consumption, as in every way humane, wise, and expedient, this being the cause of state prevention of consumption.

*Resolved*, That a copy of these resolutions duly attested be transmitted to our representatives at Albany and to the several medical societies herein mentioned, and that we urge them to use all honorable means with diligence for the promotion of this cause.

Dr. Geo. N. Jack, of Depew, N.Y., read a paper entitled, A Brief Résumé of the Grosser Animal Nature, and its Application in Medicine. Dr. W. C. Callanan's paper on Gastro-intestinal Diseases in Children was read by title.

Dr. Ballou then introduced the newly elected president, Dr. Wm. C. Phelps, who was heartily applauded on assuming the chair. He briefly expressed his appreciation of the honor.

A vote of thanks was tendered the retiring president, Dr. Ballou.

The president appointed the following committees: On membership, Drs. W. W. Potter, chairman; G. W. McPherson and J. J. Walsh. On legislation, Drs. W. W. Potter, Ernest Wende and Edward Clark.

Adjournment followed.

